# Marked Sinus Bradycardia in a COVID‐19 Patient

**DOI:** 10.1155/cric/4893066

**Published:** 2025-12-30

**Authors:** Carlos Valladares, Adam Kaplan

**Affiliations:** ^1^ Department of Internal Medicine, Rutgers Health–Community Medical Center, Toms River, New Jersey, USA

**Keywords:** case report, COVID-19, heart rate, SARS-CoV-2, sinus bradycardia

## Abstract

**Background:**

The presence of cardiac manifestations, including bradycardia, has been described as a manifestation of coronavirus disease 2019 (COVID‐19). Our case report describes a case of sinus bradycardia secondary to possible sinus node dysfunction in an otherwise asymptomatic patient with COVID‐19.

**Case Presentation:**

We report the case of an unvaccinated 33‐year‐old female hospitalized due to bradycardia after 4 days of testing positive for COVID‐19. She presented with a 1‐day history of transitory lightheadedness and dizziness with no other associated symptoms. Sinus bradycardia was recorded on admission, heart rate 52/min that later dropped to 35/min on the second day of admission. Secondary causes of bradycardia were excluded based on the absence of relevant evidence from laboratory work‐up and echocardiographic examination.

**Decision‐Making:**

We recommend baseline ECG monitoring in hospitalized COVID‐19 patients, regardless of disease severity, to assess for potential cardiac manifestations.

**Conclusion:**

Cardiac rhythm monitoring is an essential component of staying vigilant against potential complications. It raises the question of how physicians should respond to a patient with marked bradycardia and when should they intervene?

## 1. Introduction

Wuhan, China, reported the first case of coronavirus disease 19 (COVID‐19) in December 2019. Within a few months of the emergence of SARS‐CoV‐2, the virus has rapidly spread and reached a pandemic status [1]. Despite ongoing research, much remains unknown about SARS‐CoV‐2 and its systemic effects.

The most commonly affected organ system by COVID‐19 is the pulmonary system, with the most frequent clinical manifestations including cough, dyspnea, fever, and sore throat, similar to SARS and MERS [2]. In addition to respiratory manifestations, COVID‐19 is also known to cause cardiovascular manifestations [3, 4]. There have been reports in the literature of cardiac manifestations of COVID‐19, including arrhythmias [1, 4, 5]. Nevertheless, the association between COVID‐19 infections and bradycardia has only been observed in hospitalized patients with moderate to severe COVID‐19 infections involving the lungs that required intensive care unit admissions and treatment with remdesivir or tocilizumab [4–7]. Our review of the medical literature indicates that this is the first reported association between COVID‐19 and bradycardia in an otherwise asymptomatic patient without pulmonary manifestations and who did not receive antiviral or monoclonal antibodies.

## 2. Narrative

A 33‐year‐old female presented to the emergency department (ED) with a 1‐day history of intermittent dizziness and lightheadedness. She had never received a COVID‐19 vaccine and had tested positive for COVID‐19 4 days before her admission which prompted her to come to the ED. Review of systems was negative for fever, fatigue, loss of appetite, shortness of breath, or chest discomfort. She had no past medical history or recent surgical history. She was not taking any prescribed or over‐the‐counter medications. Her family history was only significant for father, mother, and brother with Type 2 diabetes. She endorsed cigarette smoking for 15 years, 1 pack/day, and stated that she quit 2 weeks prior. She also endorses 1–2 times per week cannabis use but denies any other substance use.

**Figure 1:**
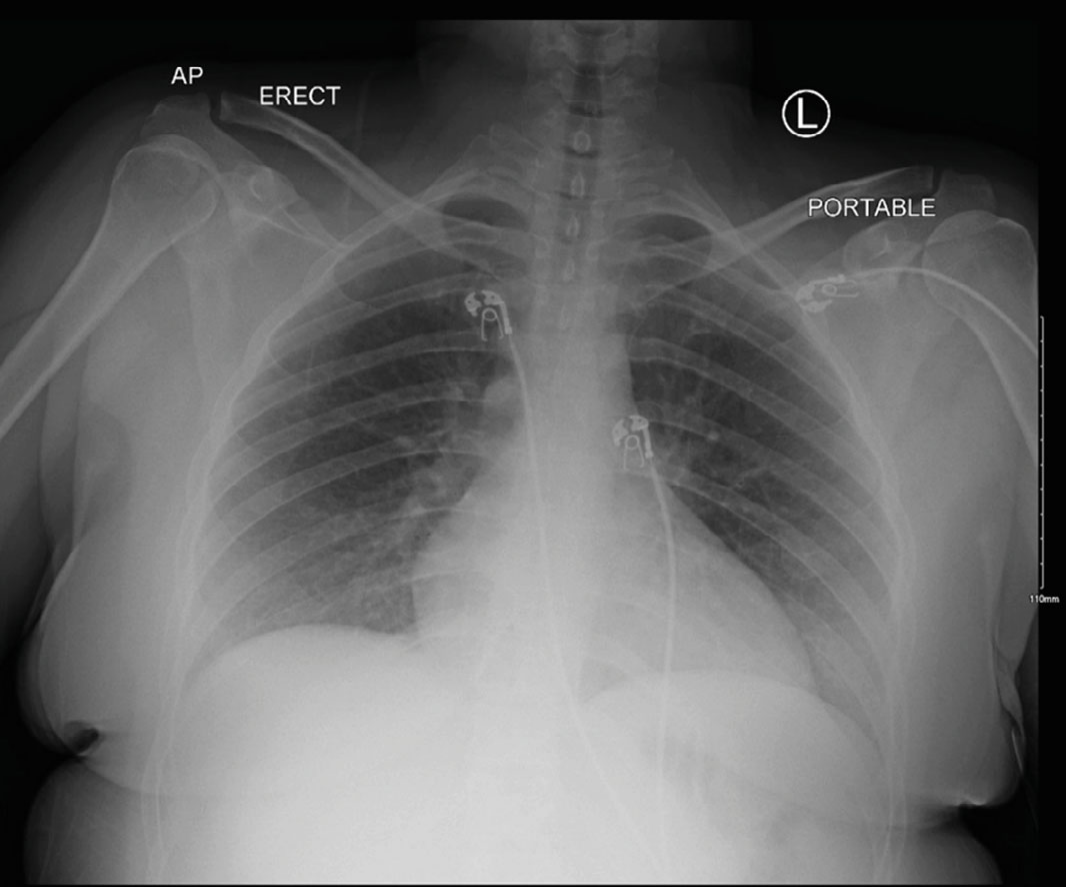
Chest x‐ray. Report: No cardiopulmonary pathologies observed.

On arrival to the ED, vitals included blood pressure of 115/61, heart rate of 59 BPM, oxygen saturation (SpO2) 99% on room air, and temperature of 97.5 F. Physical examination was unremarkable for central nervous, cardiovascular, respiratory, and gastrointestinal systems. She was alert and oriented to person, place, and time. Chest x‐ray (CXR) demonstrated no infiltrates, hyperinflation, or cardiomegaly (Figure 1).

**Figure 2:**
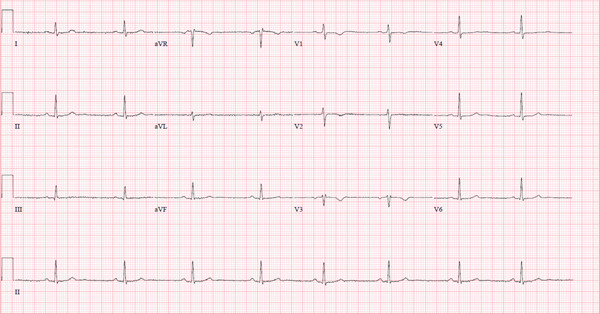
Twelve‐lead electrocardiogram showing sinus bradycardia with a heart rate of 50 BPM.

Interpretation of 12‐lead electrocardiogram (EKG) was remarkable for sinus bradycardia with a heart rate of 50 BPM (Figure 2).

Complete blood count (CBC), complete metabolic profile (CMP), cardiac profile, and TSH were ordered for further evaluation (Table 1). COVID‐19 polymerase chain reaction (PCR) test was positive. Urine drug screen was positive only for cannabis.

**Table 1:** Vital signs and relevant laboratory testing.

**Type**	**Value**	**Unit**
2022‐10‐27		
Body temperature	97.5	°F
BP (blood pressure)	115/61	mmHg
Heart rate	59	bpm
Oxygen saturation	99	%
SARS‐CoV‐2 RT‐PCR (COVID‐19)	Positive	—
Troponin	0.006	ng/mL
TSH (thyroid‐stimulating hormone)	4.98	IU/mL
2022‐10‐28		
Heart rate	44	bpm
2022‐10‐29		
Heart rate	35	bpm

**Figure 3:**
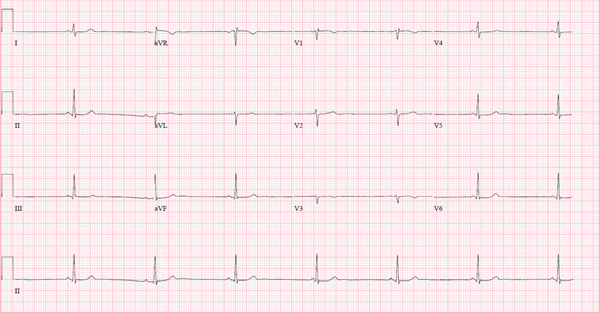
Twelve‐lead electrocardiogram showing sinus bradycardia with a heart rate of 42 BPM.

On hospital admission Day 1, telemetry monitor was ordered and recorded transient episodes of bradycardia with no other significant abnormalities. The patient was not started on any COVID‐19 specific therapy (e.g., remdesivir and Paxlovid) as she had no symptoms or risk factors. During the night, a rapid response team was activated for shortness of breath and chest pain. SpO2 was 98%, EKG revealed sinus bradycardia with short PR and heart rate of 42 BPM (Figure 3), and troponin I levels were normal. CTA was also obtained but was unremarkable.

**Figure 4:**
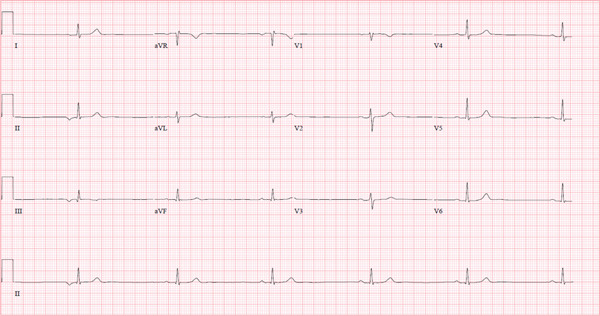
Twelve‐lead electrocardiogram showing sinus bradycardia with a heart rate of 35 BPM.

The following morning, the nurse called to report a heart rate of 32 BPM during morning rounds; another EKG was obtained that showed marked sinus bradycardia with no other findings. The heart rate recorded on the EKG was 35 BPM (Figure 4). The patient remained asymptomatic through the rest of her hospitalization course. Cardiology was consulted and performed an echocardiogram which showed normal heart structure with preserved ejection fraction.

Upon further examination, point tenderness to palpation in the left lateral rib cage was found with no skin changes. No therapeutic intervention was required at the moment. The patient continued to have transient episodes of asymptomatic bradycardia and was discharged on Day 3 of hospitalization and advised to follow up with their PCP and cardiology.

## 3. Discussion

In spite of the fact that bradycardia is not the most common symptom associated with COVID‐19 lung disease, there have been reports of patients with moderate to severe disease who developed this condition, particularly those who were taking remdesivir or tocilizumab [6]. A few studies have also indicated the potential for developing cardiac arrhythmias [4–6, 8]. Our literature review did not identify reports of sinus bradycardia in asymptomatic COVID‐19 patients who were not treated with antiviral or monoclonal therapies and had no pulmonary involvement.

There is often a correlation between the incidence of cardiovascular manifestations and the severity of the disease [9]. In our case, however, the patient′s condition was not severe and did not necessitate admission to the intensive care unit or the administration of antivirals or monoclonal antibodies. As the episode of bradycardia was asymptomatic and short‐lived, the patient received no corrective treatment. It is pertinent to note that this patient did not have any preexisting cardiac conduction abnormalities, structural heart disease, nor was she taking any AV node blocking medication that might have contributed to the bradycardia. We suspect that the episodes of bradycardia seen in our patient may partly be due to a possible dysfunction of the sinus node as a result of SARS‐CoV‐2′s direct or indirect effects on the cardiovascular system.

It has been hypothesized that COVID‐19 may cause acute bradycardia events by means of the following mechanisms: direct invasion of myocardial cells and the conduction system via angiotensin‐converting enzyme‐2 (ACE2) receptors, exacerbation of pre‐existing conduction abnormalities during acute illness, cardiac damage, hemodynamic alterations, dysfunction in the intrinsic cardiac nervous system resulting in autonomic dysfunction, a consequential effect of hypoxia induced by pulmonary injury, electrolyte abnormalities, and increased systemic inflammation [7, 8]. In addition to activating the ACE2 receptor, SARS‐CoV‐2 may also enter the host cell by utilizing the spike protein. The result of this event is a downregulation of ACE2 receptors, which has been linked to conduction disturbances following adverse myocardial remodeling [6–8].

## 4. Conclusion

Patients with COVID‐19 are increasingly presenting with bradyarrhythmia, especially those with moderate to severe disease and those on treatments such as remdesivir or tocilizumab. The specific mechanisms fundamental to the development of bradyarrhythmia in these patients remain indistinct, and they may be multifactorial [5, 6, 10]. The development of bradyarrhythmia can be considered a clinical feature of COVID‐19, which could imply cardiac involvement [6, 9, 10]. Therefore, cardiac rhythm monitoring should be performed on these patients and cardiac and inflammatory biomarker monitoring should also be considered. Regardless of the severity of the patient′s condition, we recommend a baseline ECG for patients hospitalized due to COVID‐19. Cardiac monitoring is an essential component of staying vigilant against potential complications, particularly in those with underlying cardiovascular disease, those who are elderly, and those who are taking beta‐blockers in order to ensure their safety.

This case contributes to the literature by highlighting a potential relationship between COVID‐19 and bradycardia in asymptomatic patients. Further research is needed to determine optimal management strategies for COVID‐19 patients presenting with bradyarrhythmias.

## Consent

This case report qualified for consent exemption by IRB under Category 4 (§46.101(b)(4)) in accordance with the federal policy for the protection of human subjects.

## Conflicts of Interest

The authors declare no conflicts of interest.

## Funding

No funding was received for this manuscript.

## Data Availability

The data that support the findings of this study are available from the corresponding author upon reasonable request.
